# RUNX Proteins as Epigenetic Modulators in Cancer

**DOI:** 10.3390/cells11223687

**Published:** 2022-11-20

**Authors:** Hongyang Yi, Yuhao He, Qionghua Zhu, Liang Fang

**Affiliations:** 1 Shenzhen Key Laboratory of Gene Regulation and Systems Biology, School of Life Sciences, Southern University of Science and Technology, Shenzhen 518055, China; 2 Department of Biology, School of Life Sciences, Southern University of Science and Technology, Shenzhen 518055, China; 3 National Clinical Research Centre for Infectious Diseases, The Third People’s Hospital of Shenzhen and The Second Affiliated Hospital of Southern University of Science and Technology, Shenzhen 518112, China; 4 Academy for Advanced Interdisciplinary Studies, Southern University of Science and Technology, Shenzhen 518005, China

**Keywords:** RUNX family, cancer, function, regulation, epigenetic modulator

## Abstract

RUNX proteins are highly conserved in metazoans and perform critical functions during development. Dysregulation of RUNX proteins through various molecular mechanisms facilitates the development and progression of various cancers, where different RUNX proteins show tumor type-specific functions and regulate different aspects of tumorigenesis by cross-talking with different signaling pathways such as Wnt, TGF-β, and Hippo. Molecularly, they could serve as transcription factors (TFs) to activate their direct target genes or interact with many other TFs to modulate chromatin architecture globally. Here, we review the current knowledge on the functions and regulations of RUNX proteins in different cancer types and highlight their potential role as epigenetic modulators in cancer.

## 1. Introduction

The RUNX TFs, which include RUNX1, RUNX2, and RUNX3, are master regulators of diverse developmental processes such as neurogenesis, hematopoiesis, and osteogenesis [1,2,3]. Over the past few decades, there has been a great advance in understanding the function of the RUNX family in cancer, suggesting its members as promising targets for cancer diagnosis and therapy [1,2,4,5,6,7]. Each member of the RUNX family is directly involved in multiple stages of tumorigenesis, such as the tumor formation, proliferation, apoptosis, and metastasis [1,8,9,10,11,12,13,14,15,16,17,18,19]. RUNX1 inactivation promotes the occurrence of hematological malignancies and plays a pivotal role in skin [13], breast [20,21], liver [22], and colorectal cancers [23,24]. The direct involvement of RUNX2 in tumorigenesis has been rarely reported, while the function of RUNX2 in regulating epithelial to mesenchymal transition (EMT) [19,25] and drug resistance [26] in cancer has been recently reported. RUNX3 exerts a dual function in cancer. On the one hand, it functions as a tumor suppressor in liver [27,28], lung [29,30,31,32], breast [33,34], and pancreatic cancer [9,26,35]; on the other hand, its expression level is negatively correlated with survival in head and neck squamous cell carcinomas (HNSCC) [12,36]. RUNX proteins not only activate their direct target genes, but also interact with many other TFs involved in various signaling pathways to modulate the development and progression of cancer [24,37,38,39], such as the ability of RUNX3 to directly interact and suppress the activity of the β-catenin/TCF4 complex [40,41]. In addition to acting as critical TFs, recent studies have revealed that RUNX proteins could act as pioneer factors in cancer. Our recent study showed that RUNX2 could act as a pioneer factor to modulate the epigenetic landscape to promote the EMT program [19]. This review summarizes the current knowledge on the functions and regulations of RUNX proteins in different cancer types and highlights their role as potential epigenetic modulators in cancer.

## 2. The RUNX Family Proteins

**Figure 1:**
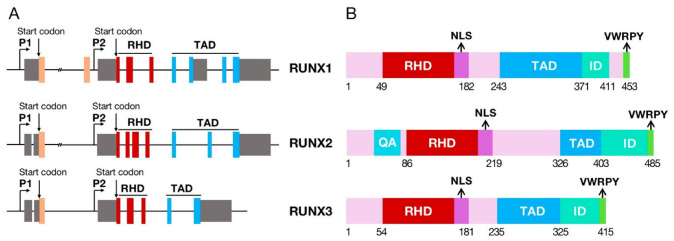
Structures of RUNX1, RUNX2, and RUNX3 genes and proteins. (**A**) Genetic structures of RUNXs. P1 (distal) and P2 (proximal) promoters are shown. Both Runt homology domain (RHD, red) and transactivation domain (TADs, blue) are present in RUNX proteins. Start codons are indicated by down arrows; coding sequences are shown in orange, red, and blue; and untranslated regions (UTRs) are in gray. (**B**) Protein structures of RUNXs. The amino acid sequences of Runt homology domain (RHD, red) are highly conserved among RUNX proteins. Other common domains include the nuclear localization signal (NLS, light purple), the transactivation domain (TAD, dark blue), the inhibitory domain (ID, light cyan), and the C-terminal Groucho/TLE binding site (VWRPY, green). The glutamine/alanine-rich (QA, light blue) sequence is RUNX2-specific.

In mammals, the RUNX family consists of RUNX1, RUNX2, and RUNX3, each with two alternative promoters: a distal P1 promoter and a proximal P2 promoter (Figure 1A) [2,3,42]. These transcripts encode RUNX proteins with different N-terminal sequences. The distal isoform typically starts with the Met-Ala-Ser-(Asp/Asn)-Ser motif and the proximal isoform starts with the Met-Arg-Ile-Pro-Val amino acid sequence [42]. The diversity of RUNX transcripts was further increased by alternative splicing. In general, RUNX1 has 9 exons and 12 isoforms, and RUNX2 has 9 exons and 12 isoforms, while RUNX3 has only 7 exons and 2 isoforms [2,3,43,44,45].

There is a highly conserved Runt homology domain (RHD) near the N-terminus of RUNX proteins, which contains 128 amino acids and mediates the binding of RUNX proteins to the DNA at the consensus RUNX motif ‘PyGPyGGTPy’ (Figure 1B) [3,42]. Meanwhile, RHD can mediate protein–protein interactions and nuclear localization of RUNX proteins. The C-terminus of the RUNX proteins is less conserved compared to the N-terminus and includes mainly the transactivation domains (TAD), the inhibitory domain (ID), and the VWRPY repression motif. VWRPY is a highly conserved motif which recruits the Groucho/Transducin-like enhancer protein (TLE) family co-repressor [3,46]. The genomic architecture and protein structures of the RUNX family TFs are shown in Figure 1.

## 3. Biological Functions of RUNX Proteins in Development

RUNX1 is a key regulator of hematopoietic cell development [3,47,48]. During mouse embryonic development, RUNX1 is essential for the generation of hematopoietic stem cells, and the deletion of Runx1 blocks the hematopoiesis process. It shows similar functions in zebrafish [49,50,51]. Meanwhile, recent studies have shown that RUNX1 also plays an important role in neuron development [46]. RUNX2 regulates bone formation and development mainly by regulating the expression of RANKL, OPG, and other genes [46,52,53]. Knockout of Runx2 in mice inhibits osteoblast differentiation, leading to the occurrence of osteoporosis [54]. Loss of function of RUNX2 also leads to the development of cranioclavicular hypoplasia syndrome, which is associated with impaired clavicular regeneration, abnormal tooth development, and delayed closure of cranial suture [55,56]. RUNX3 is an essential gene in embryonic development, whose knockout leads to early postnatal death [8,57]. In addition, studies have shown that RUNX3 is closely associated with the development of the nervous system, bone, and immune cells [2,42,46,58].

## 4. Contributions of RUNX Proteins to Carcinogenesis

### 4.1. The Intricacy Function of RUNX1 in Tumorigenesis

RUNX1 plays an important role in the development of the hematopoietic system and its mutations lead to the occurrence of hematological malignancies, such as acute myeloid leukemia (AML), acute lymphoid leukemia (ALL), and familial platelet disorder with a predisposition to acute myeloid leukemia (FPD/AML) [2,47,48,59,60,61]. Given that the majority of RUNX1 mutations detected in these patients are defined as loss-of-function mutations, RUNX1 is considered to function primarily as a tumor suppressor in these cancers. However, many studies have revealed that RUNX1 has cancer-promoting properties in solid tumors (Table 1). In many solid tumors, including colon, ovarian, and breast cancers, the expression level of RUNX1 is significantly increased compared to paracancerous tissue [7,22,23,24,62,63,64]. In a mouse model of skin cancer, deletion of Runx1 reduced the number of chemo-induced tumors [13,65]. In colorectal cancer, overexpression of RUNX1 promotes the ability of cells to migrate both in vivo and in vitro through activating the Wnt signaling pathway [23,24]. Poor survival in ovarian cancer patients is correlated with high RUNX1 expression levels, and depletion of RUNX1 in ovarian cancer cells elevates cisplatin sensitivity [16,66,67]. In addition, in HNSCC, reduced RUNX1 expression suppresses the ability of cells to migrate and proliferate in vitro and reduces tumor size in vivo [6,63]. In glioblastoma, inhibition of RUNX1 expression significantly abrogated the migratory and invasive ability of U-87 MG cells [62].

Interestingly, RUNX1 could act both as an oncogene and a tumor suppressor in breast cancer [20,21,64,65,75,76,77]. On the one hand, RUNX1 could promote breast cancer metastasis in vivo, where the expression of RUNX1 rises during the metastasis of tumor cells from the primary site to the distal lung [68]. Moreover, inhibiting the expression of RUNX1 significantly reduced the invasive and migratory capacity of the cells [68]. On the other hand, it was demonstrated that the depletion of RUNX1 could promote the estrogen-induced Wnt signaling pathway in luminal breast cancer cells by suppressing AXIN1 [23]. Van Bragt et al. found that the loss function of RUNX1 in the context of loss function of RB1 or TP53 may promote luminal breast carcinogenesis [20]. The tumor suppressive function of RUNX1 was also observed in hepatocellular carcinoma, where increased expression of RUNX1 could suppress cell migration and proliferation [22].

### 4.2. RUNX2 Promotes Cancer Metastasis

The major function of RUNX2 in the context of cancer is to promote cancer metastasis. In the skeleton system, RUNX2 is not only involved in the development, but also plays a crucial role in the formation and metastasis of osteosarcoma [1,14,78]. Lau et al. found that the chromosomal region of RUNX2 6p12-p21 was amplified in osteosarcoma, which was associated with the increased metastatic and tumorigenic properties and decreased survival rate [69,79,80]. In thyroid carcinoma, abolishing the expression of RUNX2 suppresses the expression of EMT and angiogenesis-related factors [25]. Breast cancer cells with higher RUNX2 expression have a stronger ability to migrate, partly due to the upregulation of EMT-related genes such as MMP2, MMP9, MMP13, BSP, etc. [15]. Meanwhile, in Vishal’s study, RUNX2 was found to promote breast cancer metastasis to bone [81]. Depletion of RUNX2 improves BMP-3b transcription by decreasing the methylation level of the BMP-3b promoter, thereby inhibiting migration and proliferation of lung cancer cell line H1299 [70]. Recently, our study showed that cells with activated Wnt signaling and EMT program showed higher RUNX2 expression in a heterogeneous colorectal cancer cell population and that overexpression of RUNX2 enhanced cell migration in colon cancer cells, as well as in many other cancer cells, including bladder, glioma, cervical, liver, and lung cancers [19]. We further revealed that RUNX2 could act as a pioneer factor to reshape the chromatin landscape globally to promote an EMT program.

RUNX2 has also been found to facilitate other aspects of tumorigenesis. In prostate cancer cells, RUNX2 activates the transcription of Survivin by binding to the Survivin promoter and subsequently inhibits cell apoptosis and enhances cell survival in vitro [82,83,84]. Ozaki et al. found that RUNX2 increased resistance to gemcitabine in pancreatic cancer cells [26].

### 4.3. Dual Roles of RUNX3 in Tumorigenesis

RUNX3 has been reported as a tumor suppressor in an increasing number of studies [8,9,33,85,86,87,88,89,90,91,92]. Loss of the chromosomal region (1p36), where RUNX3 is located, is frequently found in various cancers, such as breast, liver, lung, bowel, nerve, and pancreatic cancers [93]. Most remarkably, 20–40% of neuroblastoma cases show loss of heterozygosity at 1p36 [89]. Lee’s work has shown that Runx3^+/−^ mice spontaneously acquired lung adenomas around 18 months of age, whereas Runx3^−/−^ mice died from lung epithelial hyperplasia soon after birth [29,57]. Specific knockout of Runx3 in the lungs of adult mice resulted in dysregulated differentiation of lung epithelial cells and development of lung adenocarcinoma [30,57]. In different cancer types, such as lung, bone, bladder, and colon, RUNX3 expression was found to be suppressed by hypermethylation of the promoter region [33,73,89,94,95]. For example, *Helicobacter pylori* infection, a primary cause of gastric cancer, could lead to promoter hypermethylation of many tumor suppressor genes, including RUNX3 [1,2,3]. Increasing RUNX3 expression by demethylating its promoter could restrict the cell proliferation in gastric cancer [1,2,3]. The identification of the tumorigenic mutation R122C of RUNX3 in gastric and head and neck cancers was a significant breakthrough in understanding the mechanism how RUNX3 inhibits carcinogenesis [8]. The R122C mutation was shown to completely abrogate the function of RUNX3 as a tumor suppressor; while overexpression of RUNX3 slowed the proliferation of different cancer cells, overexpression of the R122C mutant failed to inhibit cell proliferation. Mislocalization of the RUNX3 protein in the cytoplasm also affects its tumor suppressor function, as has been reported in colorectal, breast, and gastric cancers [3,8,33,89].

In addition to its tumor suppressor function, RUNX3 can promote tumor development under special circumstances. Whittle et al. found that RUNX3 could also act as an oncogene and promote cell metastasis in pancreatic ductal adenocarcinoma [74]. Moreover, RUNX3 expression is higher in HNSCC than in normal tissues and is negatively correlated with survival in clinical settings [12,36].

## 5. The Regulation of RUNX Proteins in Cancer

### 5.1. At the Genetic Level

Somatic mutations in RUNX2 and RUNX3 are rare in different cancers. However, mutations of RUNX1 are detected in hematological malignancies frequently and less frequently in some solid tumors. In clinical studies, ~10% of AML patients were identified to have translocations near the RUNX1 chromosomal region [96,97], ~7% of esophageal cancer patients had RUNX1 deletion mutations [98], and ~4% of breast cancer patients had RUNX1 inactivating mutations (Table 2) [17]. In some hematological malignancies, RUNX1 proteins have been found to fuse with other genes, such as RUNX1-ETO fusion in 10–20% of AML patients and TEL-RUNX1 fusion in 20–25% of childhood ALL patients [65,99].

### 5.2. At the Transcriptional Level

**Figure 2:**
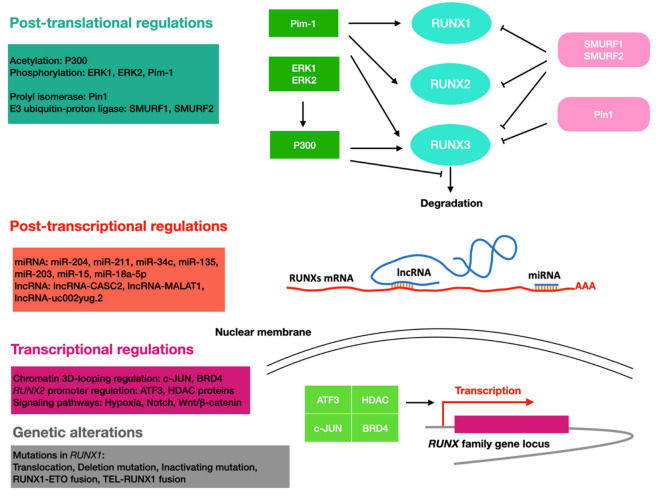
Overview of the regulation of RUNX proteins in cancer. RUNX proteins are regulated through different molecular mechanisms at different levels of gene regulation. Cyan, RUNX family proteins; red, RUNX family mRNA; purple, RUNX family gene locus; green, enzymes that induce increased levels of RUNX family proteins; pink, enzymes that induce a decrease in RUNX family protein levels; blue, ncRNAs.

The expression of RUNX can be regulated at the transcriptional level by diverse molecular mechanisms in cancer (Figure 2). In thyroid and breast cancers, RUNX2 transcription requires three distantly located enhancers (ENHs) in a chromatin three-dimensional looping. BRD4 could bind to ENHs and control RUNX2 expression, while c-JUN is pivotal for the interaction of ENHs with a set of TFs [104]. In bone metastatic breast cancer, Gokulnath et al. found that the RUNX2 promoter can be bound and activated by ATF3 [105]. In a variety of cancer cell lines, epigenetic regulators HDAC1 and other HDAC proteins could bind to the RUNX2 P2 promoter to potentiate its transcription. In thyroid cancer cells, HDAC6 could stabilize the transcriptional complex of RUNX2 [113].

RUNX proteins are also transcriptional targets of certain signaling pathways, such as Notch and Wnt/β-catenin. While RUNX1 was found to be a downstream target of the Notch signaling pathway in mouse embryonic fibroblasts and mesodermal and hematopoietic stem cells [114,115,116], RUNX3 was found to be a Notch target in endothelial cells [109]. In leukemia cell lines HL60 and Jurkat, and CD34+ progenitor cells, induction of the Wnt/β-catenin signal increases the expression of the RUNX1 P1 isoform, which may be critical for leukemia onset and progression [100]; in colon cancer cells, RUNX2 was found to be upregulated by the same pathway [19]. Hypoxia signaling is also involved in the regulation of the RUNX protein, with HIF1α and RUNX1 forming a feedback loop that fine-tunes the levels of RUNX1. In this mechanism, HIF1α transcriptionally activates RUNX1, and excess RUNX1 interacts with HIF1α and inhibits its DNA-binding and transcriptional activity [101].

### 5.3. At the Post-Transcriptional Level

RUNX can be regulated by non-coding RNAs, such as miRNA and long non-coding RNA (lncRNA). Huang et al. found that miR-204 and miR-211 could reduce RUNX2′s expression level by binding to the 3′UTR region during adipocyte differentiation [117]. It is also shown that miR-34c expression induced by P53 could reduce the expression level of RUNX2 and impair the metastatic ability of osteosarcoma cells [106]. The miR-135 and miR-203 were found to inhibit the malignant phenotypes of breast cancer cells by repressing RUNX2 [107].

LncRNA-CASC2 could antagonize miR-18a-5p-mediated repression of RUNX1, leading to reduced proliferation of multiple myeloma cells [118]. LncRNA-MALAT1 has been shown to promote colorectal cancer metastasis by interacting with RUNX2 at different steps of transcription and translation. MALAT1 could bind miR-15 to antagonize its repression on LRP6, resulting in elevated transcript levels of RUNX2 [119]. In addition, MATAL1 could bind to splicing factor SFPQ and interact with the IRES domain in the 5′UTR of RUNX2 mRNAs to elevate RUNX2 protein level [119]. LncRNA-uc002yug.2 can promote the binding of RUNX1 to an alternative splicing factor, leading to the production of more RUNX1a, thus promoting the progression of esophageal squamous cell carcinoma (ESCC) [102].

### 5.4. At the Post-Translational Level

RUNX proteins can be differentially regulated at the post-translational level, thereby affecting RUNX proteins in many aspects, such as stability, activity, and subcellular localization. For example, P300 acetyltransferase acetylates the lysine residues of RUNX1 and RUNX3. In leukemic cells, this modification was shown to increase the DNA binding ability of RUNX1 and was required for RUNX1-ETO leukemogenicity [103]. In addition, ERK1 and ERK2 phosphorylate RUNX1 in response to cytokine stimulation, thereby enhancing the interaction of RUNX1 with P300 [120,121]. The protein level of RUNX is also regulated by proteasome-mediated degradation [122]. The E3 ubiquitin–protein ligases SMURF1 and SMURF2 were found to promote RUNX degradation, while P300 acetyltransferase was shown to inhibit the degradation of RUNX3. Pim-1 could phosphorylate all RUNX proteins [123], and the phosphorylation mediated by Pim-1 would increase RUNX3′s stability and cytoplasmic location [110]. Upregulation of RUNX2 and Pim-1 was found to synergistically promote the development of T-cell lymphoma, suggesting the phosphorylation mediated by Pim-1 has a positive effect on RUNX2′s cancer-promoting function [108]. In breast cancer, the prolyl isomerase Pin1 recognizes the four phosphorylated Ser/Thr-Pro motifs in RUNX3 through its WW structural domain, thereby cis-trans-isomerizing the proline amino-terminal bond and downregulating the transcriptional activity of RUNX3. In addition, Pin1 induces ubiquitination and proteasomal degradation of RUNX3, reducing the protein level of RUNX3 in an isozyme activity-dependent manner [111].

## 6. RUNX Proteins Function as Master Regulators of Transcription

Here, we focus on discussing RUNXs’ functions as transcriptional co-regulators of different oncogenic signaling pathways and as pioneer factors capable of modulating chromatin architecture globally, aiming to highlight the role of RUNX proteins as master regulators of transcription.

### 6.1. Interaction with the Wnt/β-Catenin Signaling Pathway

Hyperactivation of the canonical Wnt signaling pathway contributes to the development and progression of various cancers [124,125]. RUNXs have a complex regulatory relationship with the Wnt signaling [19,37,41]. In hematopoietic progenitor cells and colorectal cancer cells, RUNX1 transcription is regulated by the Wnt signaling [23,100], and LEF1, a co-transcription factor of β-catenin, could interact with RUNX1 and enhance its DNA-binding ability [126]. Study has also shown that RUNX1 could directly interact with β-catenin to enhance the activity of the Wnt signaling pathway in colorectal cancer, thereby enhancing cell migration [21]. Both RUNX2 and the Wnt signaling play essential roles in osteogenesis, a highly dynamic process in which RUNX2 expression is tightly regulated by the Wnt signaling pathway. This regulatory relationship was also observed in colon cancer cells, where RUNX2 works downstream of Wnt signaling to facilitate the EMT process [19]. RUNX3 as a tumor suppressor could directly interact with the β-catenin/TCF4 complex, blocking the binding of the complex to DNA and suppressing its transcriptional activity [40,41]. This function is lost in the RUNX3 R122C mutant, which cannot interact with the β-catenin/TCF4 complex [1].

### 6.2. Interaction with the TGF-β Signaling Pathway

TGF-β signaling regulates a variety of developmental processes, and it produces conflicting phenotypes both in normal tissues and cancer. For instance, it can promote cell proliferation or apoptosis, depending on cellular context [127]. RUNX proteins are important regulators of the TGF-β signaling pathway because they can interact directly with the SMAD proteins, the TFs of TGF-β signaling, to affect the transcription of TGF-β target genes [24,74,128,129]. For instance, the interaction of RUNX3 with SMADs can induce the expression of the p21 protein and thus inhibit the cell cycle progression [10]. In hepatoma cells, RUNX1 can interact with FOXO3A to induce the transcription of BIM, a target gene of the TGF-β signaling pathway [130]. These studies demonstrated how RUNX1 and RUNX3 facilitate the tumor suppressive function of the TGF-β signaling pathway.

### 6.3. Interaction with the Hippo Signaling Pathway

Recent studies suggest that the Hippo signaling pathway contributes to carcinogenesis by directly regulating its target genes through its nuclear effectors TEAD and YAP/TAZ [38,39,131,132]. RUNX proteins could interact with different nuclear effectors of the Hippo pathway. RUNX3 can abolish TEAD binding to DNA by interacting with the N-terminal region of TEAD through its Runt domain, and this interaction can be impaired by RUNX3 R122C mutation [133]. Interestingly, although the Runt domains of RUNX1/2/3 are highly conserved, the interaction between RUNX2 and TEAD is very weak [38]. In contrast, RUNX2 strongly interacts with YAP/TAZ [134]. In breast cancer, RUNX2 promotes the nuclear localization of TAZ, but not YAP, and therefore enhances the expression of target genes [38,134]. In gastric cancer, the interaction between RUNX3 and TEAD inhibits the transcription of downstream genes, such as CTGF and CY61 [8,10,135,136].

### 6.4. RUNX Proteins Function as Pioneer Factors

Pioneer factors are defined as TFs that can directly bind the condensed chromatin and recruit other TFs and/or histone modification enzymes to activate transcription [137]. Our recent study demonstrated that by overexpressing RUNX2 alone in a cell population with low Wnt signaling activity and epithelial phenotype, RUNX2 could drive chromatin opening at most of its target loci and subsequently induce the EMT program [19]. Moreover, RUNX3 was found to bind to its target loci, where it opens chromatin structure by sequential recruitment of Trithorax group proteins and cell cycle regulators to drive cells into the restriction point, which is disrupted in nearly all tumors [138]. These studies revealed how RUNX proteins act as pioneer factors, functioning as tumor promoters and suppressors, respectively. Whether RUNX1 has a similar function in specific context remains elusive.

## 7. Conclusions

As cancer incidence and mortality rates increase, its burden on society is rapidly growing worldwide, underscoring the significance of research aimed at identifying new diagnostic and therapeutic targets. An ever-increasing number of studies have revealed the diverse functions of RUNX proteins in the formation and metastasis of various cancers, highlighting their potential as diagnostic markers or therapeutic targets [1,139,140,141,142]. Given that RUNX1 causes hematological malignancies mainly through somatic mutations and functions as a tumor suppressor, RUNX1 is more promising as a target for the treatment of solid tumors, where it is a tumor-promoting gene, with the exception of breast and liver cancers. As for RUNX2, based on its general metastasis-enhancing functions, it could be a promising target for preventing tumor metastasis and predicting patient outcomes. Given that RUNX3 is more commonly recognized as a tumor suppressor gene, it is more suitable to be developed as a diagnostic marker for solid cancers.

RUNX proteins function as epigenetic regulators in three ways: 1. They regulate the expression of their direct target genes; 2. They interact with TFs downstream of diverse signaling pathways to affect the expression of their target genes; 3. They function as pioneer factors that remodel the chromatin landscape. Given that the pioneer factor function of RUNX proteins has only recently been revealed, further studies are still required to elucidate the context specificity and detailed mechanisms of this function for different RUNX proteins, which is critical for us to understand their contributions to the physiological and pathological conditions. For instance, it would be interesting to identify in which other processes the pioneer factor function of RUNX proteins could be observed, and how RUNXs exert this function through interactions with other TFs or epigenetic modulators. Moreover, for therapeutic development, it would be of interest to test whether the pioneer factor function of RUNXs offers new possibilities for targeting specific features of cancer.

## Figures and Tables

**Table 1:** Functions of RUNX family proteins in cancer.

Gene	Cancer Type	Function	Oncogene (OG) or Tumor Suppressor Gene (TSG)	Refs
**RUNX1**	Skin cancer	promotes tumor formation	OG	[13,65]
	Colorectal cancer	promotes cell migration	OG	[23,24]
	Ovarian cancer	elevates drug resistance	OG	[16,66,67]
	HNSCC	promotes cell migration and proliferation	OG	[6,63]
	Glioblastoma	promotes cell migration and invasion	OG	[62]
	Breast cancer	promotes cell metastasis	OG	[68]
		inhibits carcinogenesis	TSG	[20]
	Hepatocellular carcinoma	inhibits cell migration	TSG	[22]
**RUNX2**	Osteosarcoma	promotes tumor formation and metastasis	OG	[1,14,69,70,71,72]
	Thyroid carcinoma	promotes EMT and angiogenesis	OG	[25]
	Lung cancer	promotes cell migration and proliferation	OG	[70]
	Breast, colon, bladder, glioma, cervical, and liver cancer	promotes cell migration	OG	[15,19]
	Pancreatic cancer	elevates drug resistant	OG	[26]
**RUNX3**	Lung cancer	inhibits carcinogenesis	TSG	[29,30,57]
	Gastric cancer	inhibits cell proliferation and metastasis	TSG	[1,2,3,71]
	HNSCC	inhibits cell proliferation	TSG	[8]
		negatively correlated with survival in clinical	OG	[12,36]
	Colorectal cancer	promotes apoptosis and inhibits metastasis	TSG	[11,72]
	Breast cancer	inhibits cell proliferation and metastasis	TSG	[73]
	Pancreatic cancer	promotes cell metastasis	OG	[74]

**Table 2:** Dysregulation of RUNX family proteins at different levels in cancer.

Gene	Level of Regulation	Type of Regulation	Cancer Type	Refs
**RUNX1**	Genetic level	Translocation	AML	[96,97]
		Deletion mutation	Esophageal cancer	[98]
		Inactivating mutation	Breast cancer	[17]
		Gene fusion	Hematological malignancies	[65,99]
	Transcriptional	Wnt signaling	Leukemia	[100]
		Hypoxia signaling		[101]
	Post-transcriptional	LncRNA-uc002yug.2	Esophageal squamous cell carcinoma	[102]
	Post-translational	Acetylation	Leukemia	[103]
**RUNX2**	Transcriptional	BRD4, c-JUN, ATF3, and HDACs	Thyroid and breast cancers	[104,105]
		Wnt signaling	Colorectal cancer	[19]
	Post-transcriptional	miR-34c	Osteosarcoma	[106]
		miR-135 and miR-203	Breast cancer	[107]
	Post-translational	Phosphorylation	T-cell lymphoma	[108]
**RUNX3**	Genetic	Deletion mutations	Breast, liver, lung, bowel, nerve, and pancreatic cancers	[93]
		Loss of heterozygosity	Neuroblastoma	[93]
		Hypermethylation	Lung, bone, bladder, colon, and gastric cancers	[1,2,3,33,73,89,94,95]
	Transcriptional	Notch signaling	Endothelial cells	[109]
	Post-translational	Phosphorylation	Breast cancer	[110]
		Ubiquitination		[111]
		Mislocalization	Breast and gastric cancers	[8,112]
